# Missing-data analysis: socio- demographic, clinical and lifestyle determinants of low response rate on self- reported psychological and nutrition related multi- item instruments in the context of the ATTICA epidemiological study

**DOI:** 10.1186/s12874-020-01038-3

**Published:** 2020-06-08

**Authors:** Thomas Tsiampalis, Demosthenes B. Panagiotakos

**Affiliations:** 1 Department of Nutrition and Dietetics, School of Health Science and Education, Harokopio University, 70 Eleftheriou Venizelou Ave., 176 71 Athens, Greece; 2 Faculty of Health, University of Canberra, Canberra, Australia

**Keywords:** Multi- item scales, Missing data, Imputation, Mediterranean diet, Depression, Low response rate

## Abstract

**Background:**

Missing data is a common problem in epidemiological studies**,** while it becomes more critical, when the missing data concern a multi-item instrument, since lack of information in even one of its items, leads to the inability to calculate the total score of the instrument. The aim was to investigate the socio-demographic, lifestyle and clinical determinants of low response rate in two self- rating multi item scales, estimating the individuals’ nutritional habits and psychological disorders, as well as, to compare different missing data handling techniques regarding the imputation of missing values in this context.

**Methods:**

The sample from ATTICA epidemiological study was used, with complete baseline information (2001–2002) regarding their demographic characteristics [*n* = 2194 subjects (1364 men: 64 years old (SD = 12 years) and 830 women: 66 years old (SD = 12 years))]. Adherence to the Mediterranean diet and depressive symptomatology were assessed at baseline, with the MedDietScore scale and the Zung’s Self- rating Depression Scale (SDS), respectively. Logistic and Poisson regression analysis were used, in order to explore the low response’s determinants in each scale. Seven missing data handling techniques were compared in terms of the estimated regression coefficients and their standard errors, under different scenarios of missingness, in the context of a multivariable logistic regression model examining the association of each scale with the participants’ likelihood of being hypertensive.

**Results:**

Older age, lower educational level, poorer health status and unhealthy lifestyle habits, were found to be significant determinants of high nonresponse rates, both in the MedDietScore scale and the Zung’s SDS. Female participants were more likely to have missing data in the items of the MedDietScore scale, while a significantly higher number of missing items in the depression scale was found for male participants. Concerning the analysis of such data, multiple imputation was found to be the most effective technique, even when the number of missing items was large.

**Conclusions:**

The present work augments prior evidence that higher non-response to health surveys is significantly affected by responders’ background characteristics, while it gives rise to research towards unrevealed paths behind this claim, especially in the era of nutritional epidemiology.

## Background

Missing data is a common phenomenon, especially, in questionnaire-based, population surveys or epidemiological studies. Presence of missing data reduce the representativeness of the selected sample, cause bias and lead to a decrease in the a-priori designed statistical power, as well as the efficiency and validity of the conducted analyses and therefore, distort inferences about the referent population [1, 2]. Although several methodological frameworks have been proposed to reduce missingness in data collection in quantitative surveys, this situation is, unfortunately, quite common in research. Moreover, it becomes more critical, especially when the missing data concern a multi-item, health-related instrument (or scale, score), which is applied to measure a latent construct that is difficult or impossible to measured directly [3]. There is a variety of such instruments that has been developed to measure psychological disorders’ symptomatology (like anxiety, depression, stress) [4], dietary patterns (like Mediterranean diet) and behaviors (like Healthy Eating pattern) [5], and several clinical conditions (like risk of developing cardiovascular disease (CVD), diabetes, obesity) [6]. Lack of information in even one of the instruments’ items, leads to the inability to calculate the total score of the instrument, making the whole procedure useless since it would not be able to correctly classify the individual to the health class belongs.

The main sources of item’s non-response are, the type of research (e.g., topic of research, referent population), the structure of the questionnaire or the instrument, the interviewer (e.g., easy acceptance of don’t’ knows (DKs)), and the background characteristics of the respondents [7–9]. Identifying the profile of individuals with missing data, is of crucial importance in order for a study and its results to be valid. For instance, individuals with missing data may be systematically different from those with complete information, either regarding the outcome of interest, or their prognosis in general. Review of the source of missingness in health surveys revealed that older individuals and low educated, as well as, females and those with poorer health status, tend to have higher levels of missing information [10].

Although several methodologies have been proposed, the aforementioned topic of missing data analysis is still not well studied and understood [11]. Complete case analysis (CCA) and proration (i.e., summing or averaging the available items with no missing data) constitute two of the most frequently used missing data handling methods [12, 13]. In spite of their simplicity, methodologists have raised several important concerns about their use, since they lead to underpowered results caused by a decreased sample size and they depend on missing data patterns and rates in the sample [14]. According to Rubin’s terminology, missing data patterns are classified as missing completely at random (MCAR) where the probability of missingness does not depend on either observed or missing data, missing at random (MAR) where conditional on the observed data, the probability of missingness is independent of unobserved data, and missing not at random (MNAR), where the probability of missingness is dependent on unobserved data even after conditioning on observed data [15].

The aim of the present work was (a) to investigate the demographic, clinical and lifestyle profile of the participants of the ATTICA epidemiological study, with missing data in two health- related scales that aimed to evaluate a psychological condition (depression) and adherence to a dietary pattern (Mediterranean diet), as well as, (b) to investigate the performance of different missing data handling methodologies on the aforementioned instruments and compare them in terms of the level on which they affect both the magnitude of the studied relationship and its uncertainty, as expressed by the standard error.

## Methods

### Sample

The working sample to test the research hypothesis of this work is the data from the ATTICA study, which is a prospective, observational cohort investigation initiated in 2001 [16]. At the baseline examination (2001–2002), *n* = 3042 apparently healthy volunteers (free of CVD and other chronic diseases) residing in the greater metropolitan Athens area, in Greece, agreed to participate (75% participation rate). Of the enrolled participants, n = 1514 (49.8%) were men [46 years old (SD = 13 years)] and n = 1528 (50.2%) were women [45 years old (SD = 14 years)]. During baseline examination, a detailed clinical evaluation was performed by trained physicians. For the purposes of this work, we excluded *n* = 848 participants with missing or incomplete demographic information and, thus the working sample consisted of *n* = 2194 subjects [1364 men: 64 years old (SD = 12 years) and 830 women: 66 years old (SD = 12 years)].

### Bioethics

ATTICA study was approved by the Bioethics Committee of Athens Medical School. The study was carried out in accordance with the Declaration of Helsinki (1989) of the World Medical Association. All participants were informed about the study aims and procedures and provided written informed consent.

### Baseline measurements

#### Socio-demographic, anthropometric and lifestyle characteristics

The socio- demographic, anthropometric and lifestyle characteristics assessed, included among others age (in years), sex (male/ female), educational level (No formal studies/ Primary education (≤ 6 years)/ Secondary education (≤ 12 years)/ Higher education (> 12 years)), body mass index (according to standard guidelines obesity was defined as body mass index > 29.9 Kg / m^2^), as well as, physical activity level (measured in MET/week) and smoking status, based on which participants were classified for the purposes of this work in two groups: Group I: Healthy lifestyle = non- smokers and physically active participants and Group II: Unhealthy lifestyle = Either smokers, or physically inactive participants.

Further details regarding the methods and measurements applied in the ATTICA study have been previously detailed [16].

### Clinical characteristics

Assessment of clinical characteristics (hypertension, hypercholesterolemia, and diabetes mellitus) was performed according to established physical examination procedures and pharmaceutical treatment [16]. In particular, diabetes mellitus was defined as a fasting blood sugar > 125 mg/dl or the use of antidiabetic medication and, thus, participants were classified as diabetic or non- diabetic. Patients whose average blood pressure levels that were measured by study’s investigators through standard procedure, were greater or equal to 140/ 90 mmHg or were under antihypertensive medication, were classified as having hypertension. Based on the total serum cholesterol levels measured, participants were classified in three groups (Group I: Desirable levels (< 200 mg/dL), Group II: Borderline levels (200–239 mg/dL) and Group III: High levels (> 240 mg/dL)), with those belonging in Group II and III, characterized as hypercholesterolemic.

### Dietary assessment

The MedDietScore, an instrument (scale) used to estimate the level of adherence to the Mediterranean diet, was applied to all participants [5]. This scale consists of 11 items estimating the frequency with which individuals consume several foods, which are either close to the Mediterranean diet (e.g., fruits, vegetables, non-refined cereals, and products), or away (e.g. meat and meat products). Higher values of this scale indicate adherence to the traditional Mediterranean diet, while lower values indicate adherence to the “Westernized” diet.

### Psychological evaluation

A translated and validated version of the Zung’s Self-Rating Depression Scale (SDS) was used, in order to assess the depressive symptoms of the participants. The scale consists of 20 items, covering affective, cognitive, and somatic symptoms, which estimate the frequency with which each symptom is experienced by the individual. Higher scores are indicative of more severe depression [17].

## Outcomes

The outcome examined in the present work was the number of missing data in the items of the two self-rating scales (i.e., MedDietScore and Zung’s SDS). Specifically, for each participant two new variables were created indicating the number of missing items in each scale. As far as the MedDietScore is concerned, participants were further classified, as those without missing data and those with missing data in at least one item, in order to investigate the characteristics of those with missing data. Concerning the Zung’s SDS, three more variables were created indicating the number of missing items in each subscale estimating the affective, cognitive, and somatic symptoms. Furthermore, in order to examine the behavior of the different missing data handling techniques with an increasing number of missing data (in each scale), participants were further classified in 3 groups, based on the number of missing items in the total MedDietScore and Zung’s SDS scale.

### Statistical analysis

Continuous variables are presented as mean values (standard deviation, SD) and categorical variables are presented as relative frequencies (%).

#### Investigation of the participants’ profile with missing data

Associations between categorical variables and the binary (no missing data/missing data in at least one item) form of the number of missing data in the MedDietScore scale, were tested with the Pearson Chi square test. Associations between the number of missing data in each scale or subscale with categorical variables, were tested with the independent samples t- test (in case of 2 categories) and the One-way ANOVA (in case of ≥3 categories). Whether these variables were normally distributed was tested through P-P plot and equality of variances through Levene’s test. Odds ratios (OR) and their corresponding 95% Confidence Intervals (95% CI) were evaluated through univariable and multivariable logistic regression analysis, which was used to find the participants’ characteristics being significantly associated with the likelihood of having missing data in at least one item of the MedDietScore scale. Incidence Rate Ratios (IRR) and their corresponding 95% CI were evaluated through univariable and multivariable Poisson regression, which was used to investigate the significant predictors of the average number of missing data in the examined scales and subscales. Backward model selection was used to determine the final significant predictors.

#### Comparison of different missing data handling techniques

Seven missing data handling methods were applied here and compared: (1) Complete case analysis (CCA), which leads to biased estimates especially when the data are MNAR, (2) Proration, which results in bias even under a MCAR mechanism, (3) Score mean imputation (SMI), (4) Item mean imputation (IMI), (5) Person mean imputation (PMI), all of which tend to cause biased estimates under every missing data mechanism (MCAR, MAR and MNAR) as the proportion of missing data increases, (6) Stochastic Regression imputation (SRI), which can yield unbiased estimates under the MAR mechanism and (7) Multiple imputation (MI), which assumes that data are MAR. After applying each method, a multivariable logistic regression model (including participants’ age and sex) was fitted, examining the association of each scale with the participants’ likelihood of being hypertensive. The aforementioned techniques were compared in terms of the estimated regression coefficients and their standard errors, while they were fitted both in the original dataset as well as, in several subsets defined by the number of missing items in the examined scales.

##### CCA

Only the subjects with complete observations for the two scales were included in the analysis, while all subjects with missing item scores were removed from the data and the model was fitted to the remaining sample.

##### Proration

Prorated scale scores were calculated for each participant, by summing the items without missing data.

##### SMI

The missing scores were imputed with the mean total score of all observed subjects.

##### IMI

A missing item score was imputed with the mean score for all complete data on that item.

##### PMI

The mean score of the items per subject was calculated, and for each subject missing item scores were imputed with this ‘personal mean score’.

##### SRI

The missing values in the total scale scores, were imputed with the regression estimates from the observed variables augmented with a normally distributed random error with a variance equal to the variance of the regression model. The regression model included as covariates the participants’ characteristics which were found to be significantly associated with the number of missing data in the two scales. In case of the Zung’s SDS the variable of hypertension was not used, since it was used as the outcome in the multivariable model for the comparison of the missing data handling techniques.

##### MI

MI was applied to the total scale scores and the imputed values were estimated from the observed variables in the dataset by an imputation algorithm and a random residual term which was added to each resulting estimate. More specifically, the imputation algorithm used was the predictive mean matching, which is appropriate for numeric data, and the imputation model included the participants’ characteristics, which were found to be significantly associated with the number of missing data in each scale. In case of the Zung SDS the variable of hypertension was not used, since it was used as the outcome in the multivariable model for the comparison of the missing data handling techniques. Finally, 5 imputed data sets were generated, which is the minimum recommended [18].

All statistical analyses were performed in the STATA software, version 14 [19], except for the SRI and MI, which were performed in R with the mice package [20].

## Results

### Sample characteristics

The mean age of the participants in the current working sample was 65 years (SD = 11.86 years), the majority of them were males (62.2%) and almost 7 out of 10 (69.5%) were at least in the secondary educational level. The prevalence of the clinical conditions studied were: 26.1% (obesity), 31.9% (diabetes), 65.6% (hypertension) and 66.9% (hypercholesterolemia), while at least 8 out of 10 participants (83.2%) were either smokers or physically inactive (unhealthy lifestyle) (Table 1).

**Table 1:** Distribution of the participants’ demographic, clinical and lifestyle characteristics, for the total sample and separately according to the level of missingness in the MedDietScore scale; the ATTICA epidemiological study

	Total sample (*N* = 2194)	No missing data (*N* = 775)	Missing data in at least one item of the MedDietScore scale (*N* = 1419)	***p***-value^*1*^	OR (95% CI)^*2*^	***p***-value^*3*^
**Demographic characteristics**						
**Age** [years; Mean (SD^*4*^)]	64.81 (11.86)	64.79 (11.77)	64.82 (11.90)	0.952	1.00 (0.99, 1.01)	0.952
**Sex** (%)				0.913		
Male	62.2	35.4	64.6		1.00	–
Female	37.8	35.2	64.8		1.01 (0.84, 1.21)	0.913
**Educational level** (%)^*5*^						
No formal studies	3.9	19.5	80.5	< 0.001	2.65 (1.14, 6.12)	0.023
Primary education	26.6	20.6	79.4		2.48 (1.60, 3.83)	< 0.001
Secondary education	55.3	27.3	72.7		1.71 (1.18, 2.48)	0.005
Higher education	14.2	39.1	60.9		1.00	–
**Clinical characteristics**						
**Obesity** (%)^*6*^						
Yes	26.1	35.9	64.1	0.956	1.01 (0.82, 1,23)	0.956
No	73.9	36.0	64.0		1.00	–
**Diabetes Mellitus** (%)^*7*^						
Yes	31.9	36.6	63.4	0.042	1.32 (1.01, 1.72)	0.042
No	68.1	43.2	56.8		1.00	–
**Hypertension** (%)^*8*^						
Yes	65.6	35.4	64.6	0.900	0.99 (0.82, 1.19)	0.900
No	34.4	35.1	64.9		1.00	–
**Hypercholesterolemia** (%)^*9*^						
Desirable levels (< 200 mg/dL)	33.1	47.2	52.8	< 0.001	0.50 (0.40, 0.62)	< 0.001
Borderline levels (200–239 mg/dL)	26.4	36.0	64.0		0.79 (0.63, 0.98)	0.048
High levels (> 240 mg/dL)	40.5	30.9	69.1		1.00	–
**Lifestyle characteristics**						
**Type of lifestyle** (%)^*10*^						
Unhealthy lifestyle	83.2	15.0	85.0	0.165	1.33 (0.89, 1.98)	0.165
Healthy lifestyle	16.8	19.0	81.0		1.00	–

**Notes**: ^*1*^*p*-value is based on the Pearson Chi- square test in case of the categorical characteristics and on the Independent samples t-test in case of the continuous characteristics. ^*2*^ OR = Odds ratio, CI = Confidence Interval. ^*3*^ p-value refers to the comparison of each category with the baseline category, while in the case of participants’ age it refers to 1- year increase. ^*4*^ SD = Standard Deviation. ^*5*^ Educational level is defined as follows: No formal studies = 0 years, Primary education≤6 years, Secondary education≤12 years, and Higher education> 12 years. ^*6*^ Obesity was defined as Body Mass Index (BMI) ≥ 29.9 kg/m^2^. ^*7*^ Diabetes mellitus was defined as a fasting blood sugar > 125 mg/dl or the use of antidiabetic medication. ^*8*^ Patients whose average blood pressure levels were greater or equal to 140 / 90 mmHg or were under antihypertensive medication were classified as hypertensives. ^*9*^ The definition of hypercholesterolemia was based on the total serum cholesterol levels. ^*10*^Healthy lifestyle = non- smokers and physically active participants, Unhealthy lifestyle = Either smokers, or physically inactive participants

### Participants’ profile with missing data

#### MedDietScore scale

**Fig. 1:**
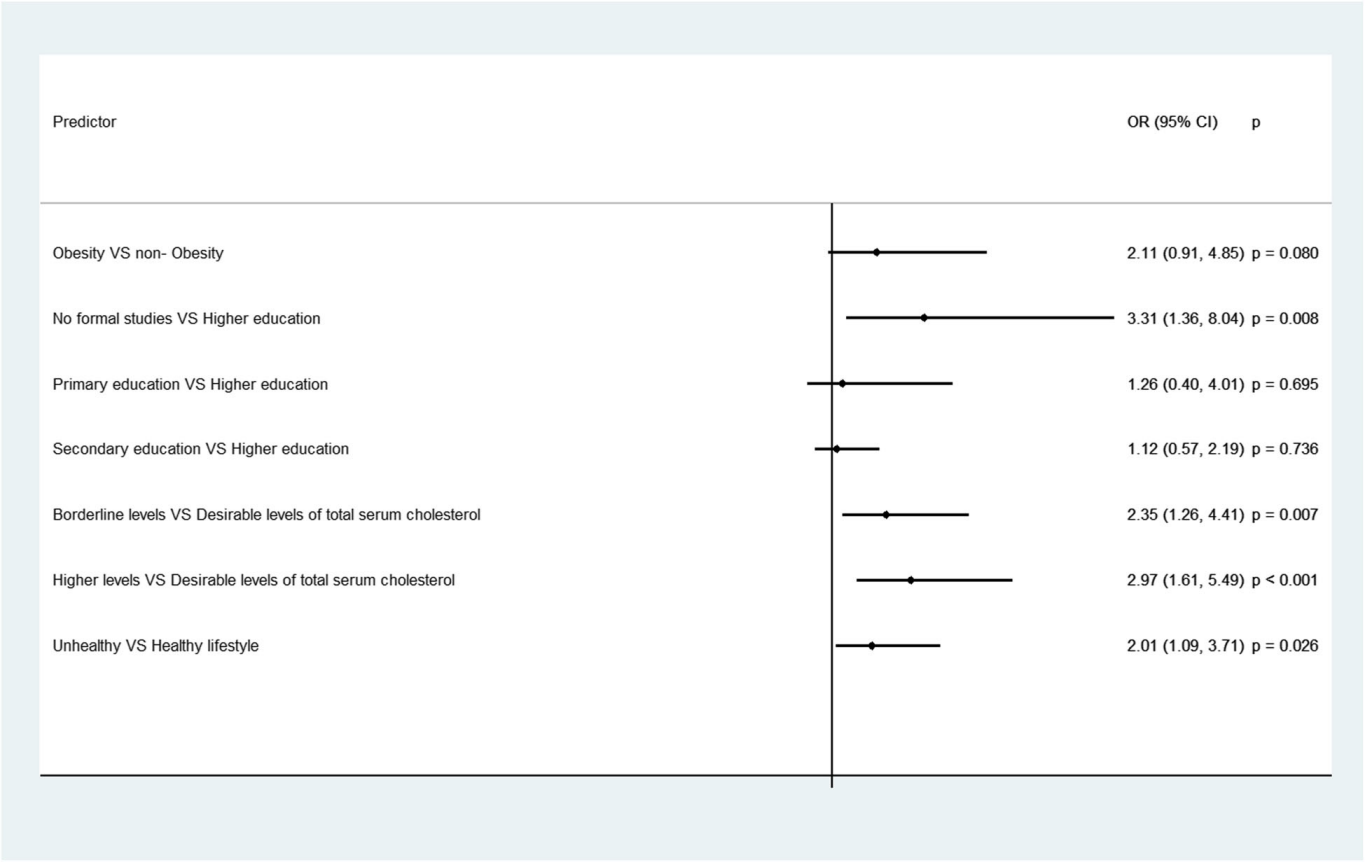
Statistically significant socio-demographic, clinical and lifestyle determinants of the participants’ likelihood of having missing data in at least one item of the MedDietScore scale; the ATTICA epidemiological study. **Notes**: Results are based on the logistic regression analysis. OR = Odds Ratio, CI = Confidence Interval. p = p-value. Educational level is defined as follows: No formal studies = 0 years, Primary education≤6 years, Secondary education≤12 years and Higher education> 12 years. Obesity was defined as Body Mass Index (BMI) ≥ 30 kg/m^2^. The definition of hypercholesterolemia was based on the total serum cholesterol levels. Healthy lifestyle = non- smokers and physically active participants, Unhealthy lifestyle = Either smokers, or physically inactive participants

**Fig. 2:**
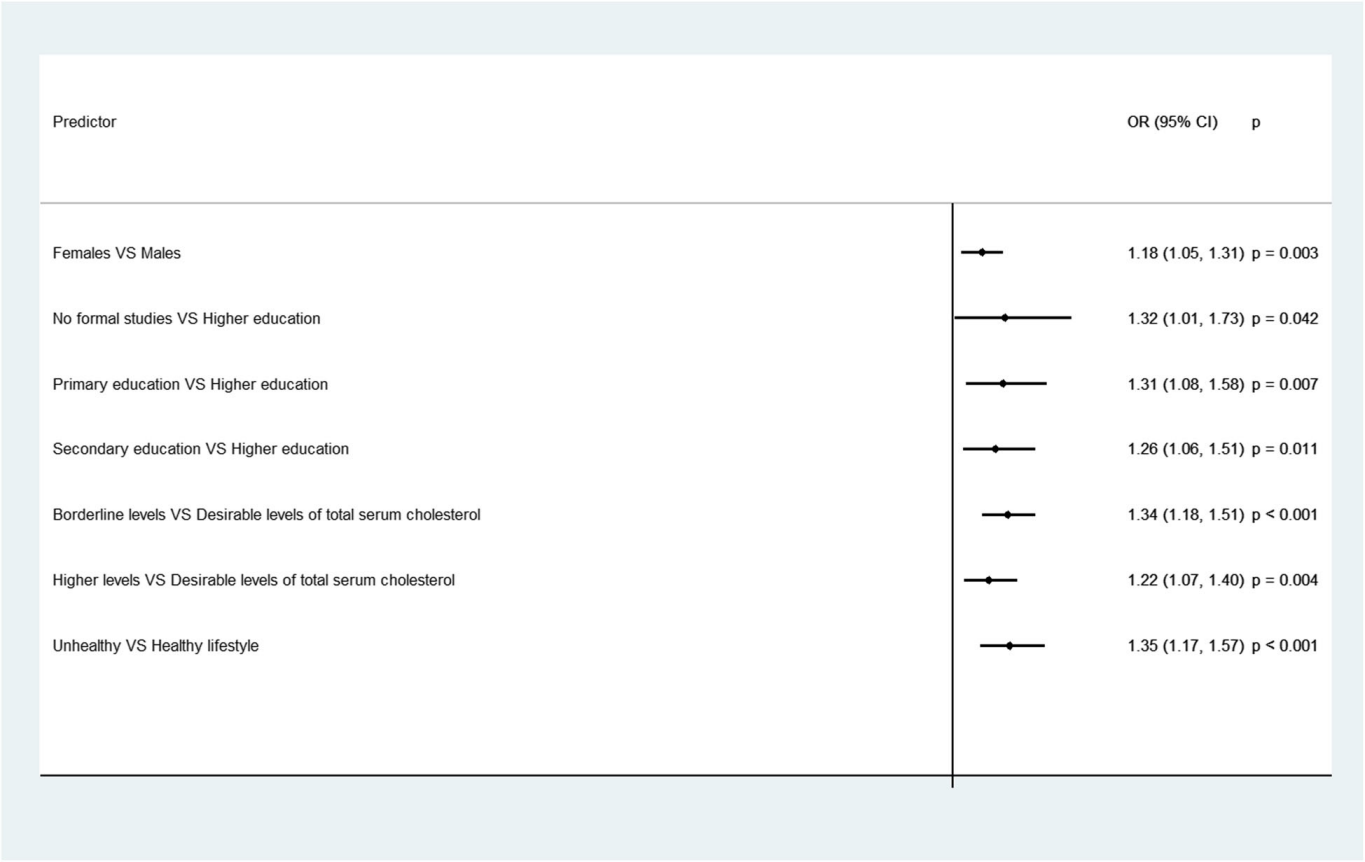
Socio-demographic, clinical and lifestyle determinants of the average number of missing items in the MedDietScore scale; the ATTICA epidemiological study. **Notes**: Results are based on the Poisson regression analysis. IRR = Incidence Rate Ratio, CI = Confidence Interval. *p* = *p*-value. Educational level is defined as follows: No formal studies = 0 years, Primary education≤6 years, Secondary education≤12 years and Higher education> 12 years. The definition of hypercholesterolemia was based on the total serum cholesterol levels. Healthy lifestyle = non- smokers and physically active participants, Unhealthy lifestyle = Either smokers, or physically inactive participants

Participants’ with missing data in at least one item of the MedDietScore scale, were less educated, more likely to be diabetic and with higher levels of total serum cholesterol, while participants following an unhealthy lifestyle seemed to have a significantly higher number of missing items in the MedDietScore scale (Tables 1, 2). Based on the results from the multivariable models, participants with missing data were more likely to be obese, to have an unhealthy lifestyle, to be less educated, with higher levels of total serum cholesterol, while female participants were also found to have a significantly higher number of missing items, when compared to males (Figs. 1, 2).

**Table 2:** Average number of missing items in the MedDietScore scale, according to the participants’ demographic, clinical and lifestyle characteristics; the ATTICA epidemiological study

	Mean (SD^*1*^)	*p*-value^*2*^	IRR (95% CI)^*3*^	*p*-value^*4*^
**Demographic characteristics**				
**Sex**				
Male	2.07 (2.65)	0.390	1.00	–
Female	2.17 (2.70)		1.05 (0.99, 1.11)	0.114
**Educational level**^*5*^				
No formal studies	2.83 (2.60)	0.002	1.61 (1.29, 2.00)	< 0.001
Primary education	2.41 (2.61)		1.37 (1.19, 1.58)	< 0.001
Secondary education	2.69 (2.90)		1.53 (1.34, 1.74)	< 0.001
Higher education	1.76 (2.41)		1.00	–
**Clinical characteristics**				
**Obesity**^*6*^				
Yes	2.05 (2.63)	0.507	1.04 (0.97, 1.12)	0.222
No	1.97 (2.58)		1.00	–
**Diabetes Mellitus**^*7*^				
Yes	1.72 (2.35)	0.007	1.31 (1.18, 1.45)	< 0.001
No	1.32 (2.05)		1.00	–
**Hypertension**^*8*^				
Yes	2.07 (2.64)	0.448	0.96 (0.90, 1.02)	0.163
No	2.16 (2.71)		1.00	–
**Hypercholesterolemia**^*9*^				
Desirable levels (< 200 mg/dL)	1.35 (2.18)	< 0.001	0.63 (0.58, 0.69)	< 0.001
Borderline levels (200–239 mg/dL)	1.89 (2.52)		0.89 (0.82, 0.96)	0.002
High levels (> 240 mg/dL)	2.13 (2.57)		1.00	–
**Lifestyle characteristics**				
**Type of lifestyle**^*10*^				
Unhealthy lifestyle	2.86 (2.78)	0.022	1.20 (1.09, 1.33)	< 0.001
Healthy lifestyle	2.38 (2.63)		1.00	–

Notes: 1 SD= Standard Deviation. 2 p-value is based on the Independent samples t-test when the categorical characteristic has two categories, and on the one-way Analysis of Variance (ANOVA) when the categorical characteristic has at least three categories. 3 IRR= Incidence Rate Ratio, CI= Confidence Interval. 4 p-value refers to the comparison of each category to the baseline category. 5 Educational level is defined as follows: No formal studies= 0 years, Primary education≤ 6 years, Secondary education≤ 12 years, and Higher education> 12 years. 6Obesity was defined as Body Mass Index (BMI)≥ 30 kg/m2. 7 Diabetes mellitus was defined as a fasting blood sugar > 125 mg/dl or the use of antidiabetic medication. 8 Patients whose average blood pressure levels were greater or equal to 140 / 90 mm Hg or were under antihypertensive medication were classified as hypertensives. 9 The definition of hypercholesterolemia was based on the total serum cholesterol levels. 10 Healthy lifestyle= non- smokers and physically active participants, Unhealthy lifestyle= Either smokers, or physically inactive participants

#### Zung’s self- rating depression scale

**Fig. 3:**
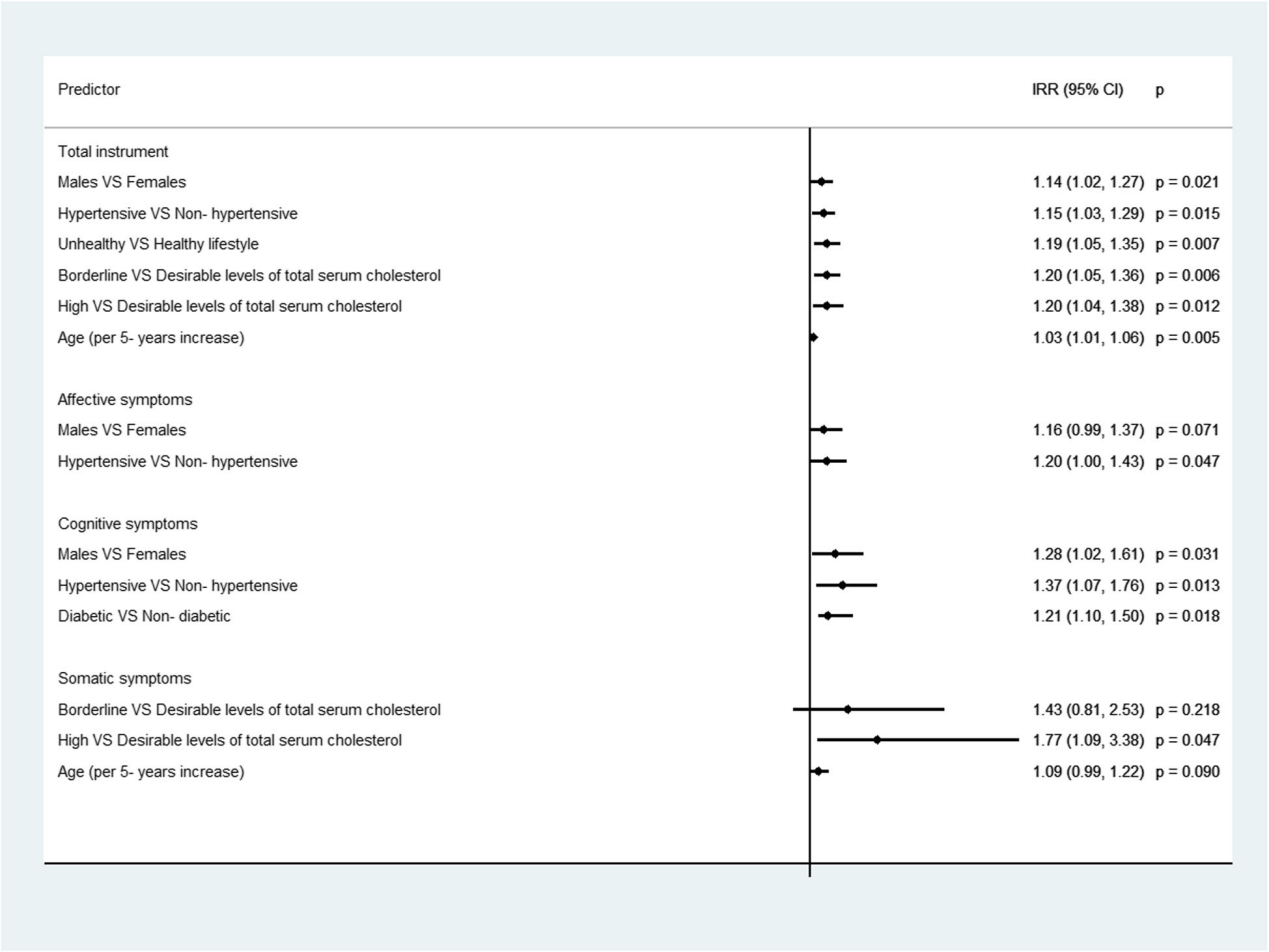
Statistically significant socio-demographic, clinical and lifestyle determinants of the average number of missing items in the total instrument Zung’s Self- Rating Depression Scale and in its subscales estimating the affective, cognitive and somatic symptoms; the ATTICA epidemiological study. **Notes**: Results are based on the Poisson regression analysis. IRR = Incidence Rate Ratio, CI = Confidence Interval. *p* = *p*-value. Educational level is defined as follows: No formal studies = 0 years, Primary education≤6 years, Secondary education≤12 years and Higher education> 12 years. The definition of hypercholesterolemia was based on the total serum cholesterol levels. Healthy lifestyle = non- smokers and physically active participants, Unhealthy lifestyle = Either smokers, or physically inactive participants. Diabetes mellitus was defined as a fasting blood sugar > 125 mg/dl or the use of antidiabetic medication. Patients whose average blood pressure levels were greater or equal to 140 / 90 mmHg or were under antihypertensive medication were classified as hypertensives

On average, participants did not respond to 8 out of 20 questions of the total instrument, with those indicating the affective symptoms having the lowest response rate, followed by the questions estimating the cognitive symptoms (Table 3). Diabetic participants had a significantly higher nonresponse rate in the total Zung’s SDS, while higher levels of total serum cholesterol were significantly associated with higher number of missing items in the three subscales. Based on the results from the multivariable Poisson regression (Fig. 3), men and older participants, those with unhealthy lifestyle, as well as, hypertensive and hypercholesterolemic participants had a significantly higher number of missing items in the total instrument, while diabetic participants had a significantly higher number of missing items in the subscale of cognitive symptoms.

**Table 3:** Average number of missing items in the total Zung Depression scale and in its sub dimensions, for the total sample and according to the participants’ demographic, clinical and lifestyle characteristics; the ATTICA epidemiological study

	Total		Affective Symptoms		Cognitive Symptoms		Somatic symptoms	
	Mean (SD^*1*^)	*p*-value^*2*^	Mean (SD)	*p*-value^*2*^	Mean (SD)	*p*-value^*2*^	Mean (SD)	*p*-value^*2*^
**Total sample**	7.97 (3.74)		3.28 (1.63)		1.85 (1.28)		0.46 (0.97)	
**Demographic characteristics**								
**Sex**								
Male	8.05 (3.71)	0.245	3.28 (1.62)	0.794	1.89 (1.27)	0.134	0.48 (0.97)	0.281
Female	7.86 (3.80)		3.27 (1.64)		1.80 (1.30)		0.43 (0.97)	
**Educational level**^*3*^								
No formal studies	7.91 (3.69)	0.351	3.32 (1.64)	0.069	1.85 (1.27)	0.112	0.57 (1.03)	0.089
Primary education	7.52 (3.74)		3.15 (1.68)		1.68 (1.27)		0.41 (0.91)	
Secondary education	7.51 (3.94)		3.13 (1.61)		1.68 (1.32)		0.39 (0.89)	
Higher education	7.29 (3.73)		2.96 (1.59)		1.54 (1.40)		0.22 (0.69)	
**Clinical characteristics**								
**Obesity**^*4*^								
Yes	8.06 (3.87)	0.515	3.28 (1.63)	0.870	1.86 (1.30)	0.840	0.52 (1.05)	0.113
No	7.94 (3.72)		3.26 (1.62)		1.85 (1.28)		0.44 (0.94)	
**Diabetes Mellitus**^*5*^								
Yes	8.33 (3.52)	0.004	3.47 (1.59)	0.002	1.97 (1.24)	0.009	0.43 (0.91)	0.564
No	7.64 (3.71)		3.15 (1.63)		1.75 (1.30)		0.39 (0.84)	
**Hypertension**^*6*^								
Yes	8.10 (3.74)	0.253	3.28 (1.63)	0.943	1.91 (1.28)	0.172	0.48 (0.98)	0.451
No	7.91 (3.74)		3.27 (1.62)		1.83 (1.28)		0.45 (0.96)	
**Hypercholesterolemia**^*7*^								
Desirable levels (< 200 mg/dL)	7.74 (3.82)	0.191	3.10 (1.62)	0.001	1.77 (1.28)	0.018	0.37 (0.88)	< 0.001
Borderline levels (200–239 mg/dL)	7.93 (3.82)		3.19 (1.63)		1.78 (1.31)		0.45 (0.95)	
High levels (> 240 mg/dL)	8.12 (3.65)		3.42 (1.60)		1.94 (1.27)		0.62 (1.10)	
**Lifestyle characteristics**								
**Type of lifestyle**^*8*^								
Unhealthy lifestyle	7.73 (3.67)	0.821	3.23 (1.63)	0.518	1.79 (1.29)	0.855	0.44 (0.93)	0.351
Healthy lifestyle	7.66 (3.77)		3.15 (1.63)		1.78 (1.29)		0.33 (0.88)	

**Notes**: ^*1*^*SD* Standard Deviation. ^*2*^*p*-value is based on the Independent samples t-test when the categorical characteristic has two categories, and on the one-way Analysis of Variance (ANOVA) when the categorical characteristic has at least three categories. ^*3*^ Educational level is defined as follows: No formal studies = 0 years, Primary education≤6 years, Secondary education≤12 years, and Higher education> 12 years. ^*4*^Obesity was defined as Body Mass Index (BMI) ≥ 29.9 kg/m^2^. ^*5*^ Diabetes mellitus was defined as a fasting blood sugar > 125 mg/dl or the use of antidiabetic medication. ^*6*^ Patients whose average blood pressure levels were greater or equal to 140 / 90 mmHg or were under antihypertensive medication were classified as hypertensives. ^*7*^ The definition of hypercholesterolemia was based on the total serum cholesterol levels. ^*8*^ Healthy lifestyle = non- smokers and physically active participants, Unhealthy lifestyle = Either smokers, or physically inactive participants

### Comparison of missing data handling techniques

#### MedDietScore scale

**Fig. 4:**
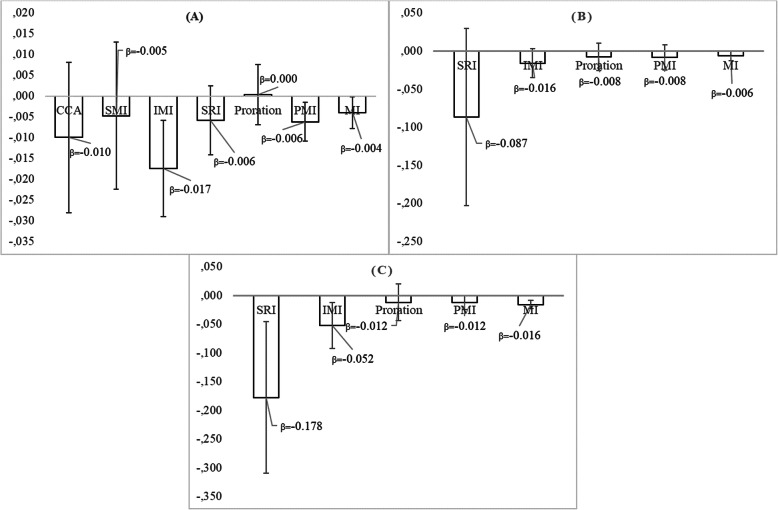
Beta- coefficient and standard error with regard to the effect of the MedDietScore on the participants’ likelihood of being hypertensive, after adjusting for age and sex, under three different scenarios concerning the number of missing items in the total scale and 7 different missing data handling methods; the ATTICA epidemiological study. **Notes**: **a** Original dataset: *N* = 1419 participants (64.7%) have missing data in the total score of the MedDietScore scale, **b** N = 855 participants have missing data in 1–2 items of the MedDietScore scale and **c***N* = 564 participants have missing data in 3+ items of the MedDietScore scale. CCA = Complete case analysis (based on 775 participants), SMI = Score mean imputation, IMI = Item mean imputation, SRI = Stochastic regression imputation, PMI = Person mean imputation, MI = Multiple imputation

In Fig. 4, the beta- coefficient with its standard error is presented, with regard to the effect of the MedDietScore variable on the likelihood of hypertension, after adjusting for participants’ age and sex. In all three cases (original data set, missing data in 9–18% of the items and missing data in > 27% of the items), the beta- coefficient of the MedDietScore variable did not differ significantly, after applying each missing data handling technique. However, after applying the multiple imputation, the standard error of the coefficient was the lowest, while after applying the stochastic regression imputation, the standard error was the highest. In the original dataset, complete case analysis led to the highest standard error and multiple imputation to the lowest.

#### Zung’s self- rating depression scale

**Fig. 5:**
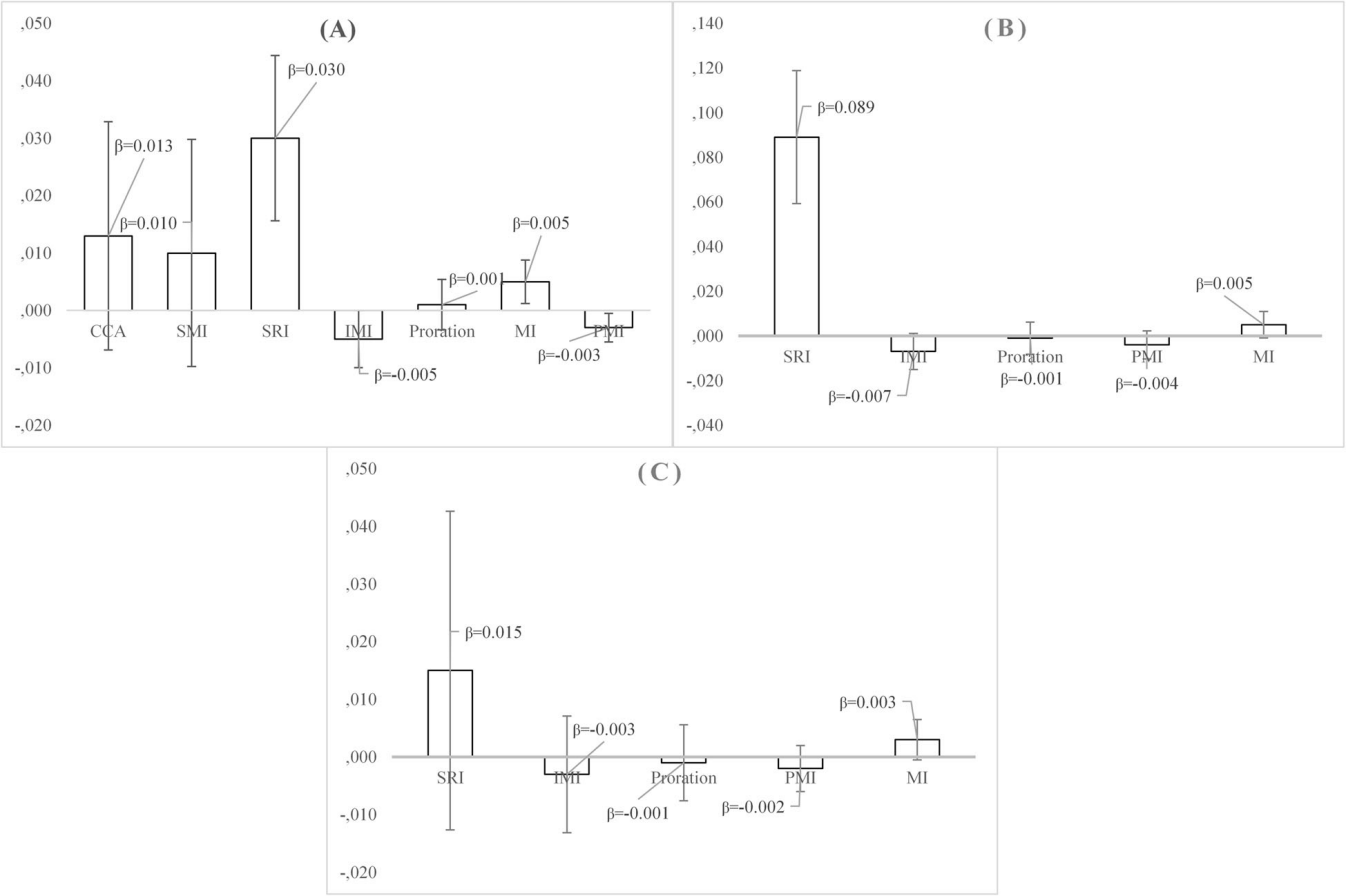
Beta- coefficient and standard error with regard to the effect of the Zung’s Self- Rating Depression Scale on the participants’ likelihood of being hypertensive, after adjusting for age and sex, under three different scenarios concerning the number of missing items in the total scale and 7 different missing data handling methods; the ATTICA epidemiological study. **Notes**: **a** Original dataset: N = 1988 participants (90.6%) have missing data in the total score of the Zung’s Self- Rating Depression scale, **b***N* = 945 participants have missing data in 1–7 items of the Zung’s Self- Rating Depression scale and (C) *N* = 1043 participants have missing data in 8+ items of the Zung’s Self- Rating Depression scale. CCA = Complete case analysis (based on 206 participants), SMI = Score mean imputation, IMI = Item mean imputation, SRI = Stochastic regression imputation, PMI = Person mean imputation, MI = Multiple imputation

Multiple imputation led to the lowest standard error, as in the previous case, when participants have missing data either in 5–35% of the items, or in > 40% of items, while in the original data set, the coefficients’ standard error was the lowest after applying the person mean imputation. As far as the beta coefficient of the Zung’s SDS variable is concerned, after applying the stochastic regression imputation, it was significantly higher when compared to the rest missing data handling techniques (Fig. 5).

## Discussion

The present work aimed to identify the profile of the individuals with missing data in two multi- item instruments, which are widely used to estimate individuals’ adherence to a healthy nutritional pattern and psychological disorders’ symptomatology, as well as, to compare some of the most widely used missing data handling techniques with regard to the efficiency and validity of the inferences. Data analyses revealed that the amount of missing data in such structured questionnaires was significantly associated with various demographic, clinical and lifestyle characteristics. In general, higher non- response rate was found to be significantly associated with older age, lower educational level, poorer health status and unhealthy lifestyle in both instruments. In addition, higher number of missing items were reported for female participants in the nutrition- related scale (MedDietScore), while the average number of missing items in the depression scale was significantly higher for male participants. Furthermore, concerning the analysis of such data, removing individuals with missing items seemed to be the worst approach, while multiple imputation was found to be the most effective method, even when the number of missing items was large. Despite the limitations of the present cross- sectional analysis, our findings revealed the profile of participants to whom special focus should be given by the researchers when collecting data, as well as, the importance of imputing the missing values in such cases.

### Participants’ profile with missing data in the MedDietScore scale

There is a substantial body of literature investigating the characteristics of the individuals with missing data in surveys, however only a small part of the research focuses on the characteristics of missing data in the context of nutritional epidemiologic studies. Our results seem to agree with those reported by Caan et al. [21], where it was found that less than 3 out of 10 participants responded to the entire questionnaire correctly, while older participants were less likely to respond correctly to the entire survey, which could be attributed to their greater susceptibility to fatigue leading them to skip food items that they do not consume. In addition, the present results are in accordance with another study, where it was reported that several lifestyle factors including age, body mass index, physical activity, and parity are significantly associated with the number of items left blank in a food frequency questionnaires (FFQ), while it was also stated that if more than 20 items on the FFQ are missing, the absolute nutrient intake may be underestimated by more than 10% [22]. Furthermore, lower educational level, as a proxy measure of the participants’ socio-economic status, was significantly associated with higher non- response in the MedDietScore scale. This result agrees with the study of Wilks et al., who were driven to the same conclusion in the context of a health survey, reporting that individuals in lower socioeconomic groups tend to present higher non- response rates in health surveys [23].

### Participants’ profile with missing data in the Zung’s SDS scale

The present findings seem to agree with the study conducted by Ying, who found that younger and higher educated men were more likely to respond to the entire instrument (Center for epidemiological studies-depression scale), while middle-aged men and older women were found to have the highest non- response rates [24]. According to Mody et al., older individuals are in a greater risk of item nonresponse by missing or skipping items, either due to cognitive impairment, or due to physical problems, such as vision impairments [25]. In addition, our finding with regard to the lower number of missing items among females, is in accordance with various previous studies reporting that female participants are more likely to participate in surveys [26–28]. Moreover, participants’ poorer health status was also connected with a higher number of missing items in the Zung’s SDS scale, which is in accordance with other studies reporting higher nonresponse rates in individuals with lower subjective health and poorer physical, cognitive, and psychological functioning [29, 30].

### Missing data handling techniques

Multiple imputation was found to be the most effective missing data handling technique in terms of the estimated standard error, either compared to the complete case analysis, or to the rest examined methods. Its efficiency over the complete case analysis could be attributed to the fact that MI uses information in the incomplete cases, to the fact that CCA is valid only in the case of MCAR data [31, 32]. Multiple imputation is a general approach which is simple to understand, but hard to program. In addition, yields unbiased estimates and provides more validity, when compared to ad-hoc approaches. Furthermore, multiple imputation was more efficient when compared to mean imputation (at the score or the person or the item level), which is a tempting but not recommended method, as it underestimates the variance in the dataset [29]. In general, our results agree with several other empirical studies in the era of nutritional epidemiology, suggesting that more advanced imputation methods, such as the MI, should be used as they give more accurate intake estimates [33–36].

### Limitations

To the best of our knowledge, this is one of the first studies investigating the profile of individuals with missing data, in such widely used instruments and to such extent. However, the conclusions of the present work should be considered under some existing limitations. First of all, the cross-sectional nature of the data does not allow for causal associations to be drawn. Another limitation is the fact that the true underlying value and the true regression coefficients of the missing data were unknown, as we did not start with a complete data set, which is a usual method in simulation studies comparing different missing data handling methods. Thus, the lack of a simulation study, that would empower the empirical data analyses, could also be considered as a methodological limitation, but this was not the purpose of the present work. Since, in the context of the present study only relative comparisons could be held among the different missing data handling techniques, we cannot conclude which imputation method is more accurate, only that the choice of method may affect both the beta coefficient of the studied relationship, as well as, its standard error. Therefore, our next step in the evaluation of the imputation methods would be to do a simulation study with a complete data set as the reference.

## Conclusions

In summary, older and less educated individuals, as well as, those with morbidities and unhealthier lifestyle habits, constitute a risk group for higher non-response rates when collecting nutrition and psychological data, and therefore, researchers should give special focus when interviewing them, in order to keep the gathered information response rate in high levels. In addition, the results from the applied data analyses revealed that the data imputation methodologies used to complete missing information, preferably the multiple imputation techniques, are trustable and may increase the validity and efficiency of the results.

## Data Availability

The data are available upon request. For expression of interest, please contact Prof. Demosthenes Panagiotakos (dbpanag@hua.gr).

## Abbreviations

CVD: Cardiovascular Disease
CCA: Complete Case Analysis
MCAR: Missing Completely At Random
MAR: Missing At Random
MNAR: Missing Not At Random
SDS: Self-rating Depression Scale
SD: Standard Deviation
CI: Confidence Interval
OR: Odds Ratio
IRR: Incidence Rate Ratio
SMI: Score Mean Imputation
IMI: Item Mean Imputation
PMI: Person Mean Imputation
SRI: Stochastic Regression Imputation
MI: Multiple Imputation
